# Fatal Necrotizing Fasciitis in a Child following a Blunt Chest Trauma

**DOI:** 10.1155/2013/373712

**Published:** 2013-03-27

**Authors:** Yohel Ocaña, Rolando Ulloa-Gutierrez, Adriana Yock-Corrales

**Affiliations:** ^1^Department of Infectious Disease, Hospital Nacional de Ninos “Dr. Carlos Saenz Herrera,” Avenida Paseo Colón, P.O. Box 1654, San José, Costa Rica; ^2^Department of Emergency, Hospital Nacional de Ninos “Dr. Carlos Saenz Herrera,” Avenida Paseo Colón, P.O. Box 1654, San José, Costa Rica

## Abstract

Necrotizing fasciitis is a serious soft tissue infection rarely occurring in children after blunt trauma. Due to its high morbidity and mortality rates, a high index of suspicion is necessary for prompt diagnosis and treatment. We describe a 6-year-old Costa Rican girl who died secondary to multiple complications following a posttraumatic necrotizing fasciitis.

## 1. Introduction

Necrotizing fasciitis (NF) is an uncommon infection of soft tissues characterized by an aggressive and rapid course that is associated with high morbidity and mortality rates [1]. Its annual incidence is 0,08 per 100,000 children [2]. We describe a patient presenting with NF after a blunt trauma of the anterior chest wall that was hospitalized and died at the only pediatric referral teaching hospital of Costa Rica.

## 2. Case Report

A 6-year-old girl presented to the emergency department (ED) of our institution with a 4-hour history of left chest pain and difficulty in breathing. Two days prior to admission, she suffered from a chest blunt trauma with a soccer ball while walking. After the impact, she fell from her height and hit the ground. She was taken immediately to the ED, where on physical examination, no skin changes or respiratory distress was found, and only tenderness of the impact area and a mild graze on the left elbow were described. On chest radiograph, the physician reported a fracture of the 2nd anterior arc rib; and she was discharged home with general recommendations.

**Figure 1:**
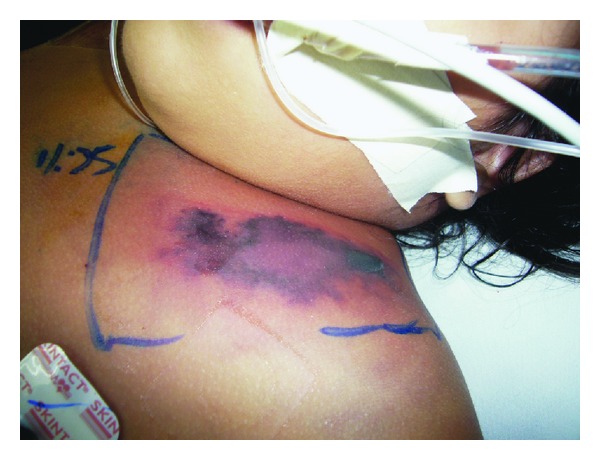
Left anterior chest wall 3 hours after admission in the emergency department. There is demarcated bruise and a necrotizing bullae.

On readmission 2 days later, both the surgical trauma and pediatric emergency teams approached the patient. Axillary temperature was 39°C, pulse rate 210/minute, respiratory rate 15/minute, and oxygen saturation on room air 81%. Noninvasive blood pressure was impossible to be obtained. She had a toxic appearance, cold and pulseless extremities, pallor skin, and Glasgow coma scale of 15. The upper left hemithorax looked edematous and violaceous (Figure 1), but no subcutaneous emphysema or crepitations were felt. No evidence of intraabdominal bleeding was detected by FAST ultrasound.

**Figure 2:**
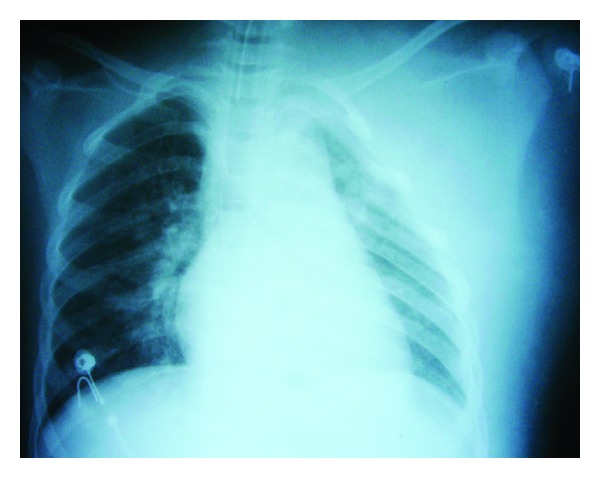
Chest radiograph following intubation showed left hemithorax soft tissue edema, decreased pulmonary expansion, pulmonary infiltrates, and no rib fractures.

**Figure 3:**
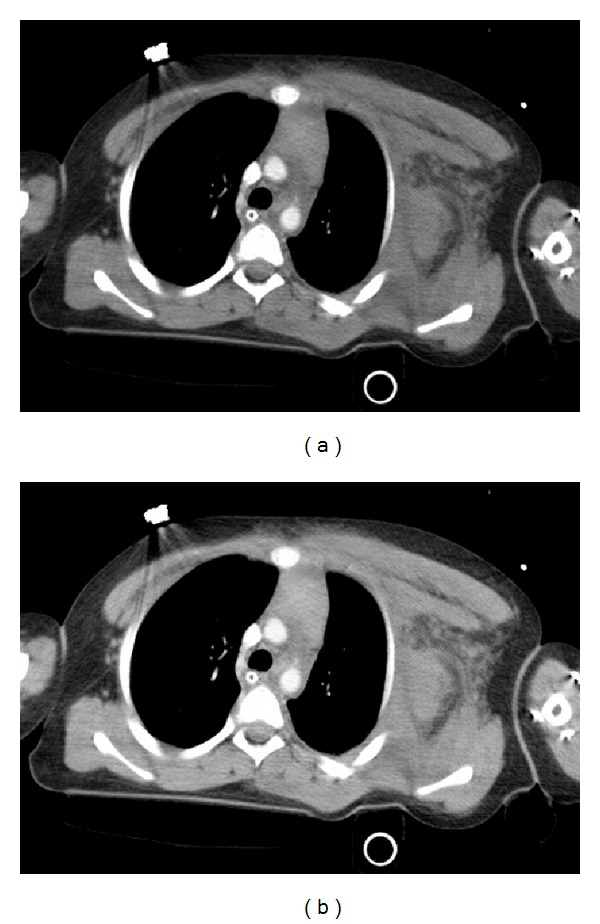
CT scan of the chest shows left apical pulmonary contusion with soft tissue edema, subcutaneous fat infiltrations, and muscular thickening with gas in the subclavicular space.

In the ED, she was intubated to protect airway and fluid resuscitation was initiated. A portable chest X-ray showed left hemithorax soft tissue edema and decreased pulmonary volume ipsilaterally (Figure 2). A chest CT revealed left apical pulmonary contusion, two fractured ribs (2nd and 3rd anterior arc ribs), and a possible collection of blood, soft tissue edema, subcutaneous fat infiltrations, and muscular thickening with gas in the subclavicular space (Figures 3(a) and 3(b)).

Laboratory investigations revealed a CBC with 22,000 leukocytes/mm^3^ (12% neutrophils, 63% band forms), hemoglobin 13 gr/dL, and platelets 82,000/mm^3^. Blood gases showed pH 7.0, pCO_2_ 71 mmHg, pO_2_ 23 mmHg, EB −12, and HCO_3_^−^ 17 mEq/L. Lactic acid was 6.9 mmol/L, BUN 28 mg/dL, creatinine 1.3 mg/dL, and CRP 120 IU/L. Two blood cultures drawn at admission from her femoral vein and subclavian CVC revealed Gram-positive cocci on stain; however, morphologically these could not be identified, no additional testing including molecular techniques was possible to obtained, and there was no bacterial growth at the end on both cultures.

**Figure 4:**
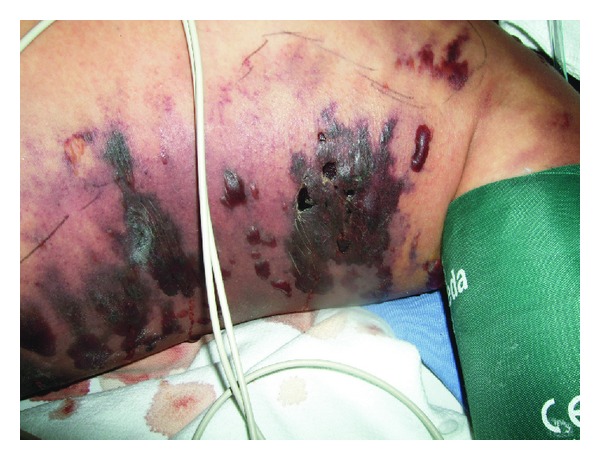
Left hemithorax 24 hours after admission to the PICU showing rapid progression of the skin changes and new lesions in the lateral chest wall and superior abdomen, hemorrhagic blisters, and severe edema and necrotic areas.

The patient underwent septic shock and multiorgan failure. In the ED, dopamine and adrenaline infusions were started after volume expansion. Intravenous cefotaxime (200 mg/kg/day) and clindamycin (40 mg/kg/day) were started in addition to multiple transfusions of fresh frozen plasma, red blood cells, and hydrocortisone. She was transferred to the PICU. Within 24 hours, the patient deteriorated significantly; progression of the skin lesions worsened (Figure 4), with new hemorrhagic blister skin lesions and severe edema and erythema extending to the left lateral chest wall. Because the patient was still very unstable, surgical debridement was on hold. She died 32 hours after PICU admission due to refractory septic shock and multiorgan failure.

## 3. Discussion

NF represents a life-threatening disease and a real challenge for clinicians because of its rapid and aggressive progression. NF is rare in children, most of the cases of NF presenting on the trunk and extremities [3].

In the pathophysiology of the disease, the microbial invasion of the subcutaneous tissues includes the superficial muscular fascia and deep dermal tissue [4]. It often occurs either through external trauma or direct spread from a break in the skin or perforated viscus. Bacteria then invade subcutaneous tissues, producing endo- and exotoxins that cause tissue ischemia, necrosis, and often systemic illness [5]. Infection can spread as fast as 1 inch per hour with little overlying skin changes [6]. There are some adult case reports of NF after blunt trauma on the chest, but none in children [7, 8].

NF is generally classified into 2 types based on the causative organism. Type 1 infections are polymicrobial with wound cultures yielding a combination of Gram-positive cocci, Gram-negative rods, and anaerobes. The antibiotic therapy should be a combination of penicillin or a cephalosporin for the Gram-positive, an aminoglycoside for the Gram-negative, and clindamycin or metronidazole for anaerobic organism [6, 9]. Type 2 NF is a monomicrobial infection, classically caused by group A  *Streptococcus*  (GAS) either alone or in association with *Staphylococcus aureus* and the antibiotic therapy should be directed accordingly [2]. Recommended intravenous antibiotic treatment for NF depends, among other factors, on the risk or possible etiologic factors, but empirically it usually involves clyndamicin, sodium penicillin, and a third generation cephalosporin.

Classic symptoms associated with NF are pain out of proportion to physical signs, violaceous coloration of the skin, anxiety, and diaphoresis. Patients might recall a history of trauma or a break in the skin within the previous 48 hours in around 10% to 40% of cases. Initially, visible skin changes can be subtle and then progress rapidly, with edema, blister and bleb formation, gangrene, and signs of compartment syndrome subsequently [2]. A high index of suspicion is needed in some of these cases to establish the diagnosis.

Associated risk factors include chronic illness, varicella, surgery, and trauma; however, a proportion of patients have no identifiable risk factors [10]. Establishing the diagnosis and giving proper therapy (medical and surgical) directly correlate with decreased mortality rates. Some authors emphasize the importance of early surgical exploration with a full thickness biopsy to confirm the diagnosis [9, 11, 12].

Radiologic studies, including plain radiographs, CT, ultrasonography, and MRI have been used to aid in diagnosing NF. These are helpful in detecting soft tissue gas and delineating the extent of damage [13, 14].

Death often occurs due to septic shock, associated complications, a delay in diagnosis and treatment, both medical and/or surgical, or a combination of all these factors. Early surgical consultation is recommended, and whenever possible, prompt surgical exploration to remove affected and necrotic tissues is recommended [9]. Other support measures include inotropic therapy, fluid resuscitation, treatment of septic shock, and management in PICU [15].

In our patient, she had an identifiable risk factor that was the blunt trauma in the chest and a mild graze on the left elbow 48 hours before presentation. The poor correlation of clinical appearance initially and the hemodynamic instability leaded to a delay in the surgical treatment. Despite aggressive medical treatment, the outcome was a refractory septic shock, multiorgan failure, and death.

## 4. Conclusion

NF is an uncommon soft tissue infection characterized by widespread fascial necrosis, often associated with severe systemic affection, usually rapid in course, and potentially fatal if not treated promptly. NF requires a high index of suspicion; prompt antibiotic therapy and surgical exploration are needed to increase survival rates. Because of its rapid progression, any soft tissue lesion should be evaluated carefully in the emergency department and initiate aggressive treatment. However, even with aggressive early medical and surgical treatment, the morbidity and mortality rates in children are high.
